# Electronic and Optical Properties of Transition-Metal-Modified BiFeO_3_: A First Principles Study

**DOI:** 10.3390/ma19010066

**Published:** 2025-12-23

**Authors:** A. P. Aslla Quispe, L. C. Huamani Aslla, B. Barzola Moscoso, M. D. Clemente Arenas, P. H. Rivera, J. D. S. Guerra

**Affiliations:** 1 Grupo de Investigación en Ciencias e Ingeniería GICI-UNIQ, Universidad Nacional Intercultural de Quillabamba, Cusco 08741, Peru; 2 Departamento de Física, Universidad Nacional de San Antonio Abad del Cusco, Cusco 08003, Peru; 155177@unsaac.edu.pe; 3 Electronics Circuits and Systems Research Group gECS-HF, Universidad Nacional Tecnológica de Lima Sur UNTELS, Villa El Salvador, Lima 15834, Peru; mclemente@untels.edu.pe; 4 Facultad de Ciencias Físicas, Universidad Nacional Mayor de San Marcos, Lima 15081, Peru; phriverar@unmsm.edu.pe; 5 Grupo de Ferroelétricos e Materiais Multifuncionais, Instituto de Física, Universidade Federal de Uberlândia, Uberlândia, Minas Gerais 38408-100, Brazil; santos@ufu.br

**Keywords:** BiFeO3, first-principles calculations, electronic properties, optical properties

## Abstract

The structural, electronic, magnetic, and optical properties are explored in the G-type antiferromagnetic BiFeO_3_ system by replacing the Fe cation with transition metals to form the BiFe_0.834_X_0.166_O_3_ compound (where X = Mn, Co, or Ni) by using first-principles DFT+U and TDDFT calculations. All the optimized structures preserve the rhombohedral (R3c) space group, showing moderate changes in the FeO_6_ octahedral distortions, lattice parameters, and Fe–O–Fe bond angles. Pristine G-type antiferromagnetic (AFM-G) BiFeO_3_ is a typical semiconductor material with a calculated bandgap energy $E_{g}=1.99$ eV. However, the inclusion of Ni, Co, and Mn at the Fe site introduces additional 3*d* states near the Fermi level, causing metallic behavior in every case. The local density of states (LDOS), density of states (DOS), and total magnetization results show that the inclusion of Ni, Co, and Mn promotes a transition from antiferromagnetic (AFM) to ferrimagnetic behavior in the modified BiFe_0.834_X_0.166_O_3_ compositions. On the other hand, in the visible spectral region, the time-dependent density functional theory (TDDFT) revealed that the pristine material has refractive index n(ω) values between 2.8 and 3.6, showing that the presence of Co and Ni enhances the extinction and absorption coefficients in both visible and ultraviolet regions, whereas the inclusion of Mn produces less significant effects. These results demonstrate that controlled substitution at the Fe site with transition metals simultaneously modifies the structural, electronic, magnetic, and optical properties of the BiFeO_3_ system, offering promising potential for applications in electronic devices with multifunctional properties.

## 1. Introduction

Bismuth ferrite (BiFeO_3_, BFO) is one of the most extensively studied multiferroic oxides over the last few decades due to the coexistence of both ferroelectric (FE) and antiferromagnetic (AFM) orders at room temperature, with Curie ($T_{C}$) and Neel ($T_{N}$) transition temperatures around 1103 K and 643 K, respectively [1,2]. Nevertheless, pristine BFO suffers from several intrinsic limitations, which include weak magnetization due to a long-wavelength spiral spin structure, the presence of leakage currents associated with oxygen vacancies, and limited absorption in the visible spectrum [3,4,5]. However, Fe-site substitution with transition metals (Co, Mn, and Ni) is one of the most effective strategies to enhance structural stability, thus improving the multifunctional performance of BFO. After replacing Fe with Mn, Co, or Ni, BFO exhibits half-metallicity, enabling the generation of highly spin-polarized currents, a characteristic that is highly favorable for applications in spintronic devices, such as spin valves, spin filters, magnetoresistive tunnel junctions, and high-efficiency MRAM memories [6]. Its robust polarization, narrow bandgap energy ($E_{g}$), lying in the 2.0–2.7 eV interval, and chemical stability make BFO a promising candidate for multifunctional applications, including photovoltaic, spintronic, and optoelectronic devices [4,5]. In order to overcome pristine BFO limitations, a widely adopted strategy has been the enhancement of the physical properties of BFO through chemical modification, with the inclusion of rare-earth elements and/or transition metals into the A- or B site, respectively, of the BFO crystalline structure. For instance, the Fe^3+^ substitution with a small concentration of Mn^3+^, Co^3+^, or Ni^3+^ cations introduces additional 3d states near the Fermi energy level, which alters the bandgap width, spin configuration, and optical properties [7]. However, at sufficient doping concentrations, conductive behavior can be induced in the BFO system, which also affects the electronic, magnetic, and optical properties of the material [8,9,10].

The major development that current technology has achieved is due to advances in both experimental and theoretical materials research. In this way, theoretical studies have been promoted in recent years by modern computer availability, as well as the implementation of new efficient computational methods, algorithms, and codes. In theoretical studies using quantum mechanics principles, we find the density functional theory (DFT) [11,12], which is an accurate method to study material properties in fundamental states. Nevertheless, in order to study material properties related to electronic and optical transitions generated by electron interactions with radiation, other approximations are commonly used. Among them, the time-dependent density functional theory (TDDFT) [13] enables describing optical properties. On the other hand, due to transition metal elements with partially filled *d* orbitals, the Hubbard potential correction in the DFT+U theory [14] has also been considered for (Fe, Mn, Co, and Ni) *d* electrons. The G-type antiferromagnetic behavior of BiFeO_3_ was modeled using two crystal sub-lattices, with Fe spin-up and spin-down electrons, respectively, giving in this case the self-consistent (SCF) calculation a total magnetization equal to zero. To study modified systems by including transition metal ions (such as Mn, Co, and Ni) instead of Fe in BFO, we first optimize the modified system to minimize energy and perform an SCF calculation to determine the modified properties of the material, such as structural, electronic, magnetic, and optical properties. The band structure is an important tool to analyze the insulate, semiconductor, or metallic behavior of the pure or modified material. The partial, local, or total density of states is used to analyze the electron’s contributions of each atom to the material properties.

At room temperature, the presence of spontaneous electric polarization in the BiFeO_3_ system indicates ferroelectric behavior, which together with the G-type antiferromagnetism provides the multiferroic character to the pure BFO, enabling this material for applications in solid-state circuits, low-energy devices, and spintronic and optoelectronic devices [15,16,17,18]. In this paper, the structural stability, band structure, magnetization, and optical properties of BiFe_0.834_X_0.166_O_3_ (X = Mn, Co, and Ni) systems are carefully analyzed, comparing their functional properties with those reported for the pure pristine G-type antiferromagnetic BiFeO_3_ system. The optical properties were analyzed in terms of the incident radiation frequency and energy, such as the dielectric functions, refraction index, reflectivity, as well as the extinction and absorption coefficients. The obtained results provide detailed insights into how the inclusion of transition metals as modifiers in the crystal structure promotes noticeable changes in the structural, electronic, and optical properties of BiFeO_3_, revealing an opportunity to expand the application of the studied materials in the design of next-generation multiferroic and optoelectronic devices [19,20].

Despite the excellent multiferroic response of BFO, the low magnetization value and limited optical window restrict its use for practical applications in optoelectronics and spintronics. In this context, the inclusion of transition metals into the Fe site promotes the reconfiguration of the electronic states near the Fermi level, modifying the band structure and some electronic properties, such as the projected and local density of states, as well as increases in magnetization, optical responses, extinction coefficient k(ω), and optical absorption α(ω). The objective is to quantify these modifications and establish a connection between the obtained changes and the functional properties, which are relevant for multiferroic, optoelectronic, and spintronic technologies.

## 2. Computational Methodology

We use first-principles calculations based on spin-polarized density functional theory (DFT) implemented in the Quantum ESPRESSO software [21,22,23]. The electron–ion interaction was described using ultrasoft pseudopotentials [24], while the electron–electron interaction was described within the generalized gradient approximation (GGA) using the exchange and correlation energy proposed by Perdew, Burke, and Ernzerhof (PBE) [25]. Due to the strongly correlated Fe-3d electrons present in the BiFeO_3_ system and partially filled *d* orbitals in the transition metal ions used as substitutional cations, the DFT+*U* correction has also been included in accordance with the linear-response approach proposed by Cococcioni and de Gironcoli [26]. The calculated Hubbard potential for the Fe-3d electrons was found to be around 3.98 eV. At the same time, for the transition metal 3d electrons used in this study (Mn, Co, and Ni), the calculated Hubbard potentials were found to be around 5.99, 4.10, and 8.88 eV for the Mn, Co, and Ni elements, respectively, consistent with the reported values in the literature [27,28]. When we modify the system by including transition metal ions (such as Mn, Co, and Ni) instead of Fe, we substitute a Fe spin-up ion with a dopant spin-up ion. Therefore, we first optimize the modified system to minimize the energy and then perform the Self-Consistent Field (SCF) calculation in order to determine the modified properties of the material, such as structural, electronic, magnetic, and optical properties.

**Figure 1:**
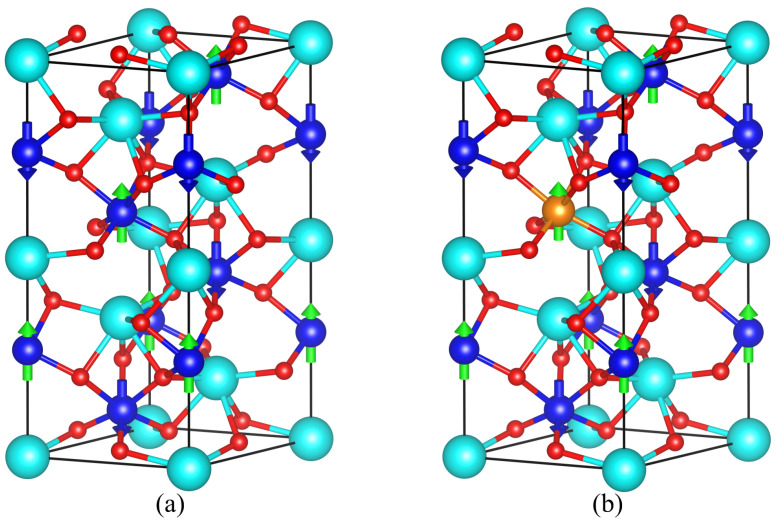
Crystal structure of (**a**) G-type antiferromagnetic BiFeO_3_, and (**b**) initial configuration for BiFe_0.834_X_0.166_O_3_.

In order to guarantee the validity of our results, first, we consider the R3c space group for the G-type antiferromagnetic BiFeO_3_ (AFM-G) and the hexagonal symmetry for the unit-cell, which is formed by 30 atoms: 6 atoms of Bi, 6 atoms of Fe, and 18 atoms of O [2,29]. For all the simulations, we performed the system optimization considering the Broyden–Fletcher–Goldfarb–Shanno (BFGS) method to minimize the total energy, allowing the unit-cell and atomic positions to change towards a configuration with the minimum total energy, as shown in Figure 1a, where the spin-up and spin-down Fe sub-lattices are represented by blue spheres with green-up and blue-down arrows, respectively. To describe the BiFe_0.834_X_0.166_O_3_ system, we substituted a Fe(↑) atom in the BiFeO_3_ structure with transition metal ions [Mn(↑), Co(↑), or Ni(↑)], as shown in Figure 1b, where the substitutional cations are represented by orange sphere with green-up arrows.

In order to resolve the Kohn–Sham equations, the BiFeO_3_ valence band is considered to be formed by 15 Bi electrons ($5d^{10}6s^{2}6p^{3}$), 16 Fe electrons ($3s^{2}3p^{6}3d^{6}4s^{2}$), and 6 O electrons ($2s^{2}2p^{4}$). For the BiFe_0.834_X_0.166_O_3_ system, we considered 15 Mn electrons ($3s^{2}3p^{6}3d^{5}4s^{2}$), 17 Co electrons ($3s^{2}3p^{6}3d^{7}4s^{2}$), and 18 Ni electrons ($3s^{2}3p^{6}3d^{8}4s^{2}$) according to the involved transition metal element. The self-consistent solution was performed using 52 Ry kinetic energy cutoff for wavefunctions, 572 Ry kinetic energy for charge density, and 6×6×6 Monkhorst–Pack [30] k-point mesh in the first Brillouin zone. The total magnetization was calculated from the difference between the *spin-up* and *spin-down* electron density of states (DOS), and the band structures and density-of-state results were used to calculate the bandgap energy and dielectric properties. On the other hand, the structural optimization was performed using $10^{-8}$ Ry total-energy convergence threshold. The kinetic energy cutoff for plane waves was set to 40 Ry and the charge-density cutoff to 320 Ry, and the equilibrium BiFeO_3_ structural parameters were obtained by fitting E(V) to the Birch–Murnaghan equation of state [31]. The optical properties were evaluated within the TDDFT implemented in the Quantum ESPRESSO software, based on the Liouville–Lanczos approach [32,33], which provides the results of the dielectric functions as a function of frequency. Using the dielectric functions, we calculate the refraction index n(ω), extinction coefficient k(ω), reflectivity R(ω), and absorption coefficient α(ω) in the 0–50 eV radiation energy range [34,35].

## 3. Results and Discussion

### 3.1. Structural Properties

The optimized crystal structure for the G-type antiferromagnetic BiFeO_3_ with R3c space group is shown in Figure 1a, represented for a hexagonal unit-cell, where the calculated lattice parameters (*a* and *b*) were found to be around 5.49 Å and 13.38 Å, respectively, in agreement with experimental and theoretical reported values in the literature [2,36]. In Figure 1a, the Bi, O, and Fe atoms are represented by sky-blue, red, and blue spheres, respectively, and the Fe spin-up and spin-down directions are indicated by green-upward and blue-downward arrows, respectively. To investigate the modified BiFe_0.834_X_0.166_O_3_ systems, we use the unit-cell shown in Figure 1b as the initial configuration, where Fe spin-up atoms on the site $0.3333\vec{a}+0.6666\vec{b}+0.3971\vec{c}$ have been replaced by X (Mn, Co, or Ni) spin-up ions, which corresponds to a transition metal concentration of x=0.166; all the other atoms remained in their equilibrium positions. In each studied case, we found that, after the optimization process, the crystal symmetry is maintained (that is to say, the crystal angles do not change), which indicates that the R3c space group remains after the substitutional process.

The summarized data in Table 1 show that the lattice parameters change due to the size of the substitutional ions as well as the change in the chemical bonds with their neighboring ions. For the BiFe_0.834_Mn_0.166_O_3_ system, we found that the *a* and *c* lattice parameters increased by 0.9% (0.05 Å) and 1.6% (0.22 Å), respectively, with respect to the pure BiFeO_3_, which promoted an increase of around 3.7% (12.78 Å^3^) in the unit-cell volume. Regarding the FeO_6_ octahedral distortions, the presence of Mn induces a decrease in the Fe–O–Fe angle from 152.58° in the BiFeO_3_ to 151.41° in the Mn–O–Fe angle for the modified system. In the case of the BiFe_0.834_Co_0.166_O_3_ system, we found that the *a* and *c* lattice parameters decrease around 0.7% (0.04 Å) and 0.5% (0.06 Å), respectively, which causes a decrease of about 1.8% (6.32 Å^3^) in the unit-cell volume with respect to pure BFO. In this case, the FeO_6_ octahedral distortions revealed that the substitution of Fe by Co induces an increase in the Fe–O–Fe angle from 152.58° (in the BiFeO_3_) to 152.65° in the Co–O–Fe angle of the modified system. On the other hand, the calculated parameters for the BiFe_0.834_Ni_0.166_O_3_-optimized system showed that, similar to the BiFe_0.834_Co_0.166_O_3_ composition, the space group is R3c, and the *a* and *c* lattice parameters decreased around 1.8% (0.10 Å) and 4.1% (0.55 Å), respectively, leading to a decrease of 7.3% (25.58 Å^3^) in the unit-cell volume with respect to the BiFeO_3_ system. For this case, the FeO_6_ octahedral distortions evidenced that the substitution of Fe by Ni induces an increase in the Fe–O–Fe angle from 152.58° (in BiFeO_3_) to 153.12° in the Ni–O–Fe angle of the modified system.

In summary, the structural data shown in Table 1 confirm that the BiFe_0.834_X_0.166_O_3_ (X = Mn, Co, or Ni) crystal structure maintains the R3c space group of BiFeO_3_, and the changes in the structural parameters are directly associated with the properties of each transition metal ion used to replace Fe^3+^ (with 0.64 Å ionic radius). For instance, the replacement of Fe^3+^ with Mn^3+^ with a higher ionic radius (around 0.65 Å) promotes an increase in the lattice parameters and, therefore, in the unit-cell volume. On the other hand, the substitution of Fe^3+^ by Co^3+^ or Ni^3+^, with ionic radii of around 0.61 Å and 0.56 Å, respectively, leads to a decrease in the optimized lattice parameters and unit-cell volume.

### 3.2. Electronic Properties

**Figure 2:**
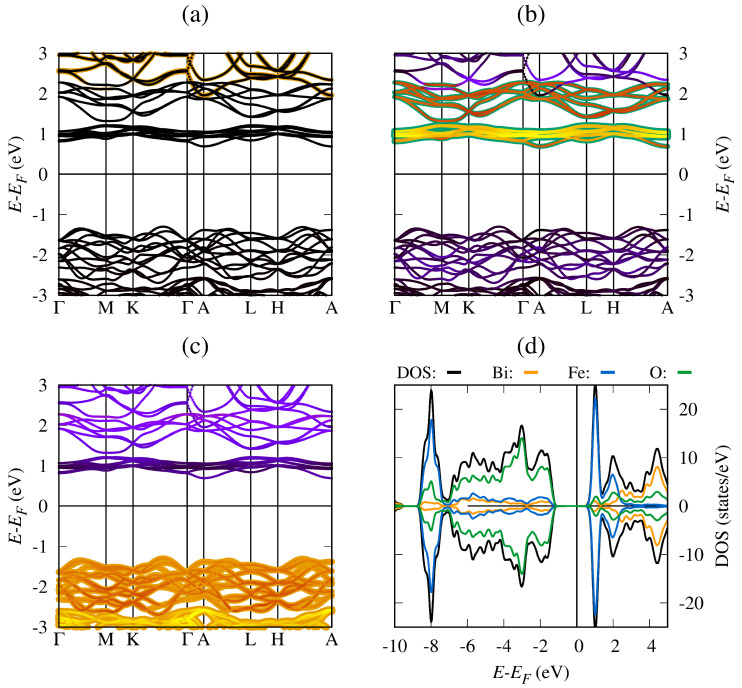
Band structure and density of states for BiFeO_3_ (**a**) fat-bands with Bi(↑↓) PDOS, (**b**) fat-bands with Fe(↑↓) PDOS, (**c**) fat-bands with O(↑↓) PDOS, and (**d**) LDOS and DOS.

For the study of the electronic properties, we performed a self-consistent solution of the Kohn–Sham equations using the optimized system to finally evaluate the band structure and density of states. For the G-type antiferromagnetic BiFeO_3_ (BFO), Figure 2 shows the band structure and density of states for the G-type antiferromagnetic BFO around the Fermi energy level, represented by $E-E_{F}=0$ eV, and using the high-symmetry points Γ–*M*–*K*–Γ–*A*–*L*–*H*–*A* in the first Brillouin zone. The calculated indirect bandgap energy was found to be around 1.99 eV between the Γ point in the valence band and the *M* point in the conduction band; this bandgap value is in agreement with previous DFT+U reports [37,38] and confirms the semiconductor behavior of the material. Figure 2a includes the local density of states (LDOS) associated with bismuth (Bi) atoms near the Fermi energy. In the valence bands, the Bi-6*s* electrons are important for chemical bonds and structural stability, while the Bi-6*p* unoccupied states are important for the conduction band and electronic transitions. However, near the Fermi energy, the Bi-5*d* electron contribution is negligible. Figure 2b shows the Fe electron contribution to the BiFeO_3_ band structure, the Fe(↑↓)-3*d* states being the most important near the Fermi energy level. The electronic transitions from the valence band to the conduction band occur preferentially due to the Fe(↑↓)-3*d* states, located around $E-E_{F}=1.0$ eV. Figure 2c shows oxygen’s contribution to the crystal structure stability and the chemical bonds, the O-2*p* states being the most important near the Fermi energy level, particularly in the valence band with maximum LDOS around $E-E_{F}=-3.0$ eV.

Regarding density of states, Figure 2d shows the LDOS and DOS for the pure BiFeO_3_ between $E-E_{F}=-10.0$ eV and $E-E_{F}=5.0$ eV, where the Bi, Fe, or O LDOS is represented by orange, blue, or green lines, respectively. The BiFeO_3_ total DOS is represented by black lines. The results confirm the semiconductor behavior of the material and indicate that, near the Fermi energy level, the valence band is formed mainly by O-2*p* and Fe-3*d* hybridization. In contrast, in the conduction band near the Fermi level, the conduction bands are mainly formed by Fe-3*d* and Bi-6*p* hybridization [27,39]. The symmetry of the LDOS and DOS graphs for spin-up (↑) and spin-down (↓) electrons confirms the zero total magnetization of BFO, which is in agreement with the antiferromagnetic character of the pristine system [40].

**Figure 3:**
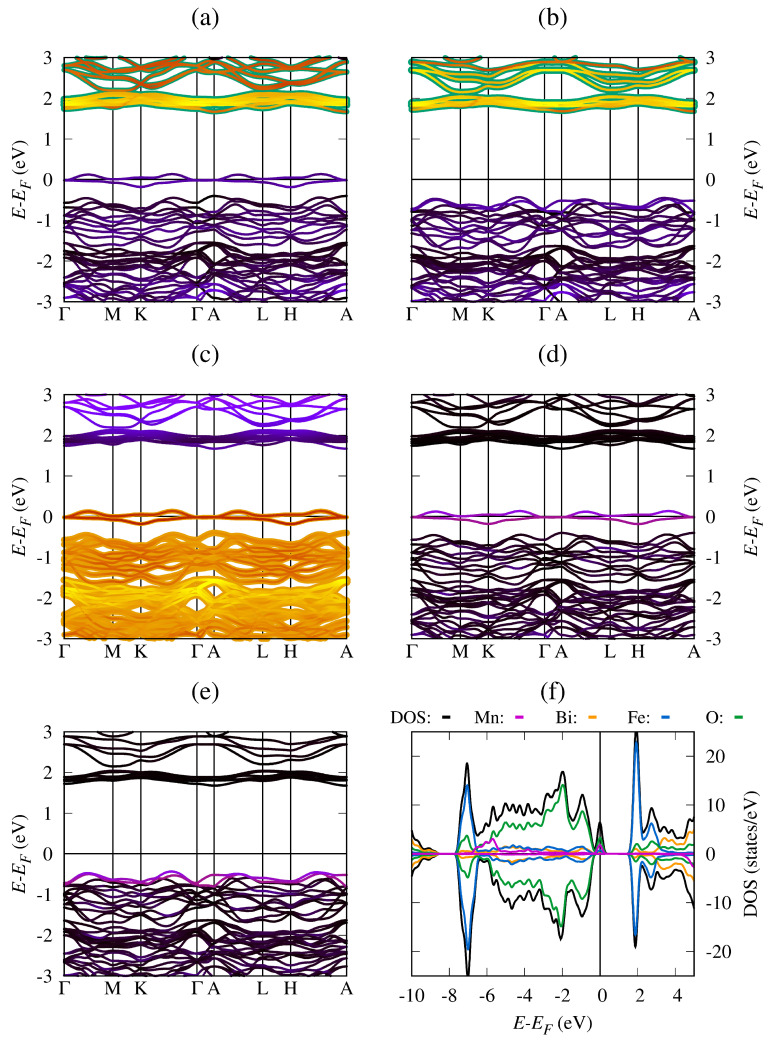
Band structure and density of states for the BiFe_0.834_Mn_0.166_O_3_ system (**a**) fat-bands with Fe(↑) PDOS, (**b**) fat-bands with Fe(↓) PDOS, (**c**) fat-bands with O(↑) PDOS, (**d**) fat-bands with Mn(↑) PDOS, (**e**) fat-bands with Mn(↓) PDOS, and (**f**) LDOS and DOS.

Figure 3 shows the band structure, LDOS, and DOS for the BiFe_0.834_Mn_0.166_O_3_ system, calculated using the energy levels and wave functions obtained via a self-consistent solution of the Kohn–Sham equations. Figure 3a shows a high localization of the Fe(↑)-3*d* unoccupied states (1.0 states/eV) in the conduction band near the Fermi energy level. In addition, Figure 3b shows that the Fe(↓)-3*d* states make an important contribution, but slightly lower than the Fe(↑)-3*d* states for the same energy levels. Around the Fermi level, the Fe(↑)-3*d* states have a small contribution, while the Fe(↓)-3*d* states do not contribute. Figure 3c highlights the importance of the oxygen ions in the chemical bonds and formation of the valence band, guaranteeing crystal structure stability and the electronic properties of the material, mainly due to the high presence of O(↑)-2*p* states under the Fermi energy level. In Figure 3d, the Mn(↑)-3*d* states make a significant contribution to the occupied states (2.0 states/eV) around the Fermi energy level, which transform the semiconducting behavior of BiFeO_3_ into conductive behavior. On the other hand, in Figure 3e, the Mn(↓)-3*d* states do not contribute to the conductive behavior of the modified system; however, they are important for energy level formation below the Fermi energy level. Finally, in Figure 3f, the LDOS and DOS results confirm the half-metallic behavior of the BiFe_0.834_Mn_0.166_O_3_ system, the spin-up electrons of Mn, Fe, and oxygen being the causes of such behavior. The asymmetric behavior of the LDOS and DOS graphs reveals the ferrimagnetic nature of the modified system and justifies the presence of a non-zero total magnetization, in agreement with the ferrimagnetic state reported in the literature [41].

**Figure 4:**
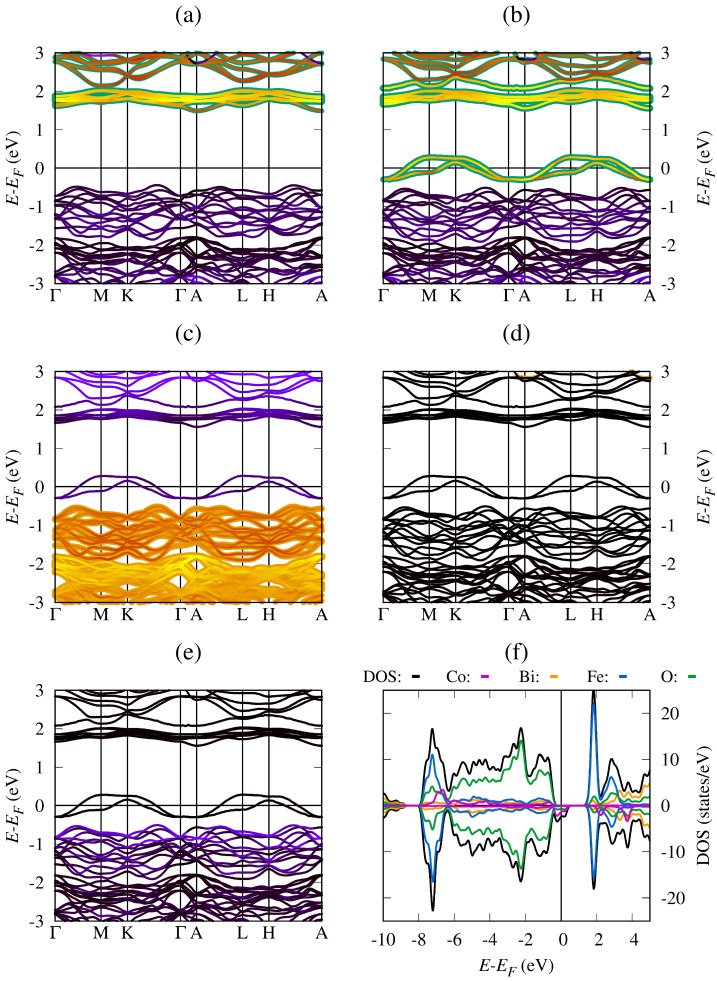
Band structure and density of states for the BiFe_0.834_Co_0.166_O_3_ system (**a**) fat-bands with Fe(↑) PDOS, (**b**) fat-bands with Fe(↓) PDOS, (**c**) fat-bands with O(↓) PDOS, (**d**) fat-bands with Bi(↓) PDOS, (**e**) fat-bands with Co(↓) PDOS, and (**f**) LDOS and DOS.

Figure 4 shows the calculated band structure for the BiFe_0.834_Co_0.166_O_3_ system obtained using the results of the self-consistent solution of the Kohn–Sham equations. Figure 4a shows a high localization of the Fe(↑)-3*d* unoccupied states in the conduction band, showing that they do not cause the conductive behavior. In contrast, in Figure 4b, we observe that the Fe(↓)-3*d* states make an important contribution to the conductive behavior around the Fermi energy level. Figure 4c shows that the oxygen ions participate in the formation of states that produce conductive behavior, but the O(↑)-2*p* states contribute more to chemical bond formation in the valence band under the Fermi energy level. Figure 4d shows that the Bi(↓)-5*d* states make a significant contribution to the occupied states around the Fermi energy level and the conductive behavior. On the other hand, the Co(↓)-3*d* states in Figure 4e contribute more to the conductive behavior of the modified system and the valence band formation below the Fermi energy level. Figure 4f shows the LDOS and DOS results, confirming the conductive behavior of the BiFe_0.834_Co_0.166_O_3_ system, the spin-down electrons of Co, Fe, and oxygen being the cause of such behavior. The asymmetric behavior of the LDOS and DOS reveals the ferrimagnetic nature of the modified system, in agreement with previous reports [42].

**Figure 5:**
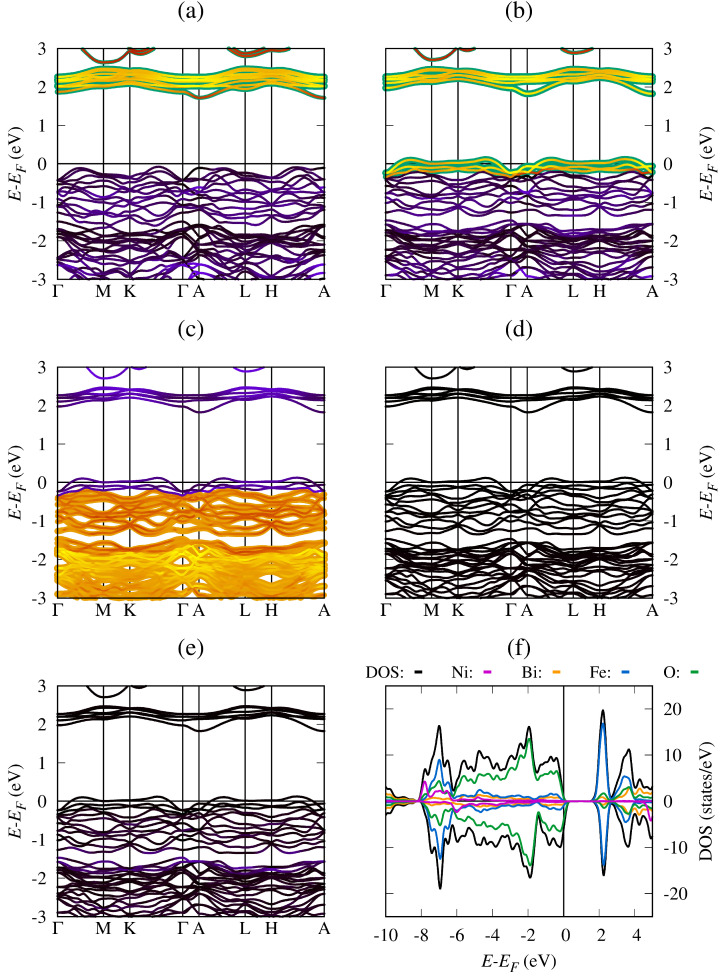
Band structure and density of states for BiFe_0.834_Ni_0.166_O_3_ system (**a**) fat-bands with Fe(↑) PDOS, (**b**) fat-bands with Fe(↓) PDOS, (**c**) fat-bands with O(↓) PDOS, (**d**) fat-bands with Bi(↓) PDOS, (**e**) fat-bands with Ni(↓) PDOS, and (**f**) LDOS and DOS.

Figure 5a shows the results for the BiFe_0.834_Ni_0.166_O_3_ system, revealing that the Fe(↑)-3*d* unoccupied states are important in the conduction band, but they do not produce conductive behavior. However, it can be seen in Figure 5b that the Fe(↓)-3*d* states contribute to conductive behavior and form states around the Fermi energy level. According to Figure 5c, the O(↑)-2*p* states contribute to the formation of states that produce conductive behavior and contribute more to chemical bond formation in the valence band under the Fermi energy level. Figure 5d shows that the Bi(↓)-5*d* states make a significant contribution to the occupied states around the Fermi energy level. On the other hand, the Ni(↓)-3*d* states in Figure 5e are important for the conductive behavior of the modified system and valence band formation. In Figure 5f, the LDOS and DOS results confirm the conductive behavior of the BiFe_0.834_Ni_0.166_O_3_ system, the spin-down electrons of Ni, Fe, and oxygen being the causes of such behavior. The asymmetric observed behavior of the LDOS and DOS reveals the ferrimagnetic nature of the modified system, consistent with previous studies [43]. In BiFe_0.834_Ni_0.166_O_3_ (X = Mn, Co, and Ni), modified compound half-metallic behavior is observed in which one spin channel remains metallic while the opposite channel is a semiconductor with 2.11 eV, 1.93 eV, and 1.79 eV energy bandgap values, respectively. The asymmetries observed in the DOS around the $E_{F}$ allow the electronic currents to exhibit spin polarization, making these systems promising candidates for spintronic devices, such as spin filters, spin valves, magnetoresistive tunnel junctions (TMR), and high-efficiency MRAM memories.

### 3.3. Magnetic Properties

For the magnetic property analysis, we use the LDOS and DOS results, calculated using the Kohn–Sham wave functions. In the G-type antiferromagnetic BiFeO_3_ case, the Fe magnetic moment by site is 4.00 μ_B_. The total magnetization calculated from the difference between the spin-up and spin-down electron density is zero, and the absolute magnetization is 25.77 μ_B_/cell, consistent with the presence of two sub-lattices with Fe(↑) spin-up and Fe(↓) spin-down, indicating the antiferromagnetic nature of the studied BiFeO_3_ system [2,40]. When we replace one Fe atom with (Mn, Co, or Ni) transition metal ions, changes in the number of electrons in partially filled *d* orbitals are observed, so the magnetic moment per site of the Mn ions in the BiFe_0.834_Mn_0.166_O_3_ system is 4.22 μ_B_. The calculated total magnetization is around −1.00 μ_B_/cell due to the higher contribution of spin-down Mn(↓) electrons with respect to the spin-up Mn(↑), around $E-E_{F}=-7.0$ eV, as shown in Figure 3f. This result and the total magnetization value of 26.66 μ_B_/cell indicate that the material has ferrimagnetic behavior, with more spin-down electrons than spin-up electrons. At the same time, when the Fe is replaced by Co to form the BiFe_0.834_Co_0.166_O_3_ system, the Co magnetic moment per site, total magnetization of the system, and absolute magnetization values are revealed to be around 2.93, −1.00 μ_B_/cell, and 24.72 μ_B_/cell, respectively. Therefore, in this case, the material also exhibits ferrimagnetic behavior. On the other hand, in the BiFe_0.834_Ni_0.166_O_3_ system, the magnetic moment per site of the Ni atoms, total magnetization of the system, and absolute magnetization values were found to be around 1.66 μ_B_, −2.15 μ_B_/cell, and 22.95 μ_B_/cell, respectively. The reported data in Table 2 reveal that all the studied modified systems exhibit ferrimagnetic behavior.

The spin multiplicity of the BFO system was obtained using the zero total magnetization, producing singlet states with spin multiplicity 1.0. The BiFe_0.834_Mn_0.166_O_3_ and BiFe_0.834_Co_0.166_O_3_ systems have total magnetization −1.00 μ_B_/cell, producing a doublet state with a spin multiplicity of 2.0. On the other hand, the BiFe_0.834_Ni_0.166_O_3_ system has a triplet state with a spin multiplicity of 3.0 due to its total magnetization of −2.15 μ_B_/cell. This $M_{T}\neq 0$ generates magnetoelectric coupling that can be used for applications in magnetoelectric sensors and spintronics.

### 3.4. Optical Properties

To analyze the optical properties, we use the wave function results of the Kohn–Sham equations, in which the TDDFT approximation is performed. The TDDFT calculations produce the dielectric function defined as $\varepsilon \left(\omega \right)=\varepsilon_{1}\left(\omega \right)+i\varepsilon_{2}\left(\omega \right)$. With these results, we evaluate the optical paremeters, such as the refractive index n(ω), the extinction coefficient k(ω), the absorption coefficient α(ω), and the reflectivity R(ω), expressed in terms of $\varepsilon_{1}\left(\omega \right)$ and $\varepsilon_{2}\left(\omega \right)$ as

$$n\left(\omega \right)=\sqrt{\frac{|\tilde{\varepsilon }\left(\omega \right)|+\varepsilon_{1}\left(\omega \right)}{2}},\;k\left(\omega \right)=\sqrt{\frac{|\tilde{\varepsilon }\left(\omega \right)|-\varepsilon_{1}\left(\omega \right)}{2}},$$

$$\alpha \left(\omega \right)=\frac{4\pi \;k(\omega )}{\lambda },\;R\left(\omega \right)={\left|\frac{\tilde{n}\left(\omega \right)-1}{\tilde{n}\left(\omega \right)+1}\right|}^{2},$$

where n(ω) is defined as the ratio of the velocity of light in a vacuum relative to the velocity of light passing through that material, and k(ω) represents the degree of photon absorption as it passes through the material. The α(ω) describes the ratio of photon intensity loss per unit length due to absorption, R(ω) indicates the ratio between the energy of reflected photon and the energy of incident photon on a surface, and $|\tilde{\varepsilon }\left(\omega \right)|=\sqrt{\varepsilon_{1}^{2}\left(\omega \right)+\varepsilon_{2}^{2}\left(\omega \right)}$.

**Figure 6:**
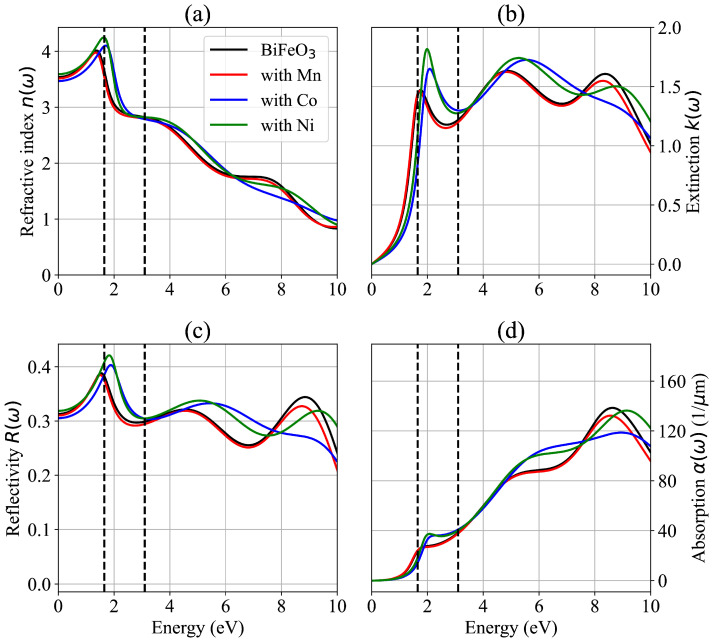
Calculated optical properties of the G-type BiFeO_3_ and modified systems, where the vertical traced lines indicate the visible spectra limits. (**a**) Refractive index n(ω), (**b**) extinction coefficient k(ω), (**c**) reflectivity R(ω), and (**d**) absorption coefficient α(ω).

Figure 6 shows the calculated optical properties of BiFeO_3_ with hexagonal symmetry obtained using the TDDFT theory [34,35]. The obtained results are consistent with the experimental and theoretical results previously reported [41,44,45,46]. The vertical lines in Figure 6 represent the limits of the visible spectrum, which in radiation energy lies in the 1.65–3.10 eV range, corresponding to the wavelength of 400–750 nm. As shown in Figure 6a for the G-type antiferromagnetic BiFeO_3_, the n(ω) has a maximum value of around 4.02 for a photon energy of 1.37 eV in the infrared spectrum. In the visible spectrum, n(ω) changes from 3.60 (at 1.65 eV) to 2.83 (at 3.10 eV). When we replace the Fe with the Mn ion, the n(ω) has a maximum value of 3.98 for a photon energy of around 1.34 eV in the infrared spectrum, showing a shift of around 0.03 eV relative to the n(ω) maximum of the BiFeO_3_ system. In the visible spectrum, n(ω) changes from 3.53 (at 1.65 eV) to 2.83 (at 3.10 eV). In the BiFe_0.834_Co_0.166_O_3_ system, the n(ω) maximum value is found to be 4.10 for a photon energy of 1.70 eV in the visible spectrum, showing a shift of 0.57 eV with respect to the n(ω) maximum of the BiFeO_3_ system. For this case, in the visible spectrum, n(ω) changes from 3.53 (at 1.65 eV) to 2.83 (at 3.10 eV). For the BiFe_0.834_Ni_0.166_O_3_ system, the n(ω) maximum value is 4.25 for a photon energy of 1.63 eV in the infrared spectrum, showing a shift of 0.55 eV with respect to the n(ω) maximum of the BiFeO_3_ system.

Figure 6b shows the extinction coefficient k(ω), where it can be seen that, for the G-type BiFeO_3_ system, the k(ω) increases in the infrared spectrum from zero to reach a maximum value of 1.47 for a photon energy of 1.75 eV in the visible spectrum. On the other hand, we observe that k(ω) has a minimum value in the visible spectrum that occurs for a photon with energy of around 2.69 eV. When we replace the Fe with the Mn cation, we find that k(ω) has a maximum of 1.46 (at 1.72 eV) in the visible spectrum, which represents a shift of 0.03 eV compared to the case of BiFeO_3_. The blue lines show that, in the visible region, the replacement of Fe by Co causes an increase in k(ω), indicating higher attenuation of the photon in the visible spectrum, with a maximum value of 1.65 at 2.08 eV. Similarly, the green lines show that, when Fe is replaced by Ni, k(ω) reaches a maximum value of 1.82 for the energy 1.99 eV in the visible spectrum, also indicating higher photon absorption compared to the other studied cases.

Regarding the reflectivity results, Figure 6c shows that R(ω) has values higher than 0.30 in the infrared spectrum. The pure BiFeO_3_ has a maximum value of 0.39 at 1.54 eV in the IR range and a minimum value of 0.30 at 2.81 eV in the visible spectrum. The red curves indicate that the presence of Mn instead of Fe produces very small changes in the infrared spectrum, with a maximum shift of 0.03 eV toward lower energies. The minimum in the visible spectrum also shifts toward lower energies in 0.05 eV and reaches a value of 0.29 at 2.75 eV. Unlike the replacement of Fe with Mn, when we replace Fe with Co (blue line) or Ni (green line), we observe that the maximum of R(ω) shifts toward positive energies in the visible spectrum, with values of 0.40 (at 1.88 eV) and 0.42 (at 1.82 eV), respectively.

Figure 6d depicts the absorption coefficient and reveals that α(ω) has small values for all the studied cases in the infrared spectrum, being smaller for cases of replacing Fe with Co and Ni compared to the pure BiFeO_3_ and BiFe_0.834_Mn_0.166_O_3_ systems. In contrast, in the visible spectrum, the α(ω) coefficient for the BiFe_0.834_Co_0.166_O_3_ and BiFe_0.834_Ni_0.166_O_3_ compositions increases to higher values than those observed for BiFe_0.834_Mn_0.166_O_3_ and BiFeO_3_. In the UV region, between 3.5 eV and 4.65 eV photon energies, the observed behavior for α(ω) is similar. However, we can observe large differences in α(ω) for photon energies higher than 4.65 eV. These results are in agreement with experimental data reported in the literature [46,47,48].

Therefore, it can be seen that the substitution of Fe by Mn, Co, and Ni transition metals in the G-type antiferromagnetic BiFeO_3_ substantially modifies the optical properties, the changes caused by the Co and Ni ions being higher than the changes promoted by the Mn ion. The ability to tune these properties through doping is of great interest for optimizing photonic devices, photocatalysis, sensors, spintronics, actuators, and optoelectronic applications [49]. These increases in k(ω) and α(ω) under Co/Ni doping enable applications such as photodetectors, modulators, and integrable spectral filters, and the dispersion of n(ω) allows for the design of waveguides/delay lines in integrated photonics.

## 4. Conclusions

In this study, the density functional theory, DFT+U, and TDDFT were systematically employed to elucidate the structural, electronic, magnetic, and optical properties of G-type antiferromagnetic BFO with the R3c space group, where the system is modified by replacing the Fe ion with another transition metal element (Mn, Co, or Ni). After crystal structure optimization, the results reveal that the modified system conserves the *R3c* space group in every case. However, substantial changes in octahedral distortions have been observed, revealed by the changes in the Fe–O–Fe angles, which correlate with the ionic radius of the substitutional ions (Mn, Co, or Ni) and the electronic configuration of the 3*d* orbitals. For the pure BFO system, we find that the semiconductor behavior is retained, with a calculated bandgap energy of 1.99 eV and valence band near the Fermi level formed mainly by the hybridization of O-2*p* and Fe-3*d* states, and the lowest energy levels of the conduction band are predominantly constituted by Fe-3*d* and Bi-6*p* states. The density of states and magnetization results confirm the antiferromagnetic behavior of the studied BiFeO_3_. The substitutional ions induce the appearance of 3*d* states associated with the transition metal in the vicinity of the Fermi level, which modify the behavior of BiFeO_3_ to half-metallic behavior in the three studied cases (with Mn, Co, and Ni), generating asymmetry in the spin-up and spin-down electron density of states. This effect produces a total magnetization different from zero, showing the transition to ferrimagnetic behavior of the modified systems. Regarding the optical properties, the results reveal that the presence of Co and Ni increases both k(ω) and α(ω) coefficients in the visible–UV region, enabling additional transitions and broadening the spectral range for applications. Furthermore, the inclusion of Mn produces minor changes, which are advantageous when relative transparency is desired. In the pure BiFeO_3_ system, n(ω) varies from 3.6 to 2.8 in the visible spectrum, and α(ω) reaches values of up to 140 $\mu m^{-1}$ in the UV spectrum. The obtained results provide an atomistic view of the properties in the modified material, which is essential for the design of electronic devices that use magnetoelectric coupling and electronic and optical properties for applications in photocatalysis, integrated photonics, and spintronics.

## Figures and Tables

**Table 1:** Optimized structural parameters for the pure BiFeO_3_ and the BiFe_0.834_X_0.166_O_3_ systems.

System	*a* (Å)	*c* (Å)	X–O–Fe Angle	Volume (Å)^3^
BiFeO_3_	5.49	13.38	152.58	348.78
BiFe_0.834_Mn_0.166_O_3_	5.54	13.60	151.41	361.56
BiFe_0.834_Co_0.166_O_3_	5.45	13.32	152.65	342.46
BiFe_0.834_Ni_0.166_O_3_	5.39	12.83	153.12	323.20

**Table 2:** Magnetic properties for the BiFeO_3_ and BiFe_0.834_X_0.166_O_3_ systems.

System	Magnetic Moment (μ_B_)	$M_{t}$ (μ_B_)/Cell	|M| (μ_B_)/Cell
BiFeO_3_	Fe →4.00	0.00	25.77
BiFe_0.834_Mn_0.166_O_3_	Mn →4.22	−1.00	26.66
BiFe_0.834_Co_0.166_O_3_	Co →2.93	−1.00	24.72
BiFe_0.834_Ni_0.166_O_3_	Ni →1.66	−2.15	22.95

## Data Availability

The original contributions presented in this study are included in the article. Further inquiries can be directed to the corresponding author.
